# Meta-Analysis of Alterations in Regulatory T Cells' Frequency and Suppressive Capacity in Patients with Vitiligo

**DOI:** 10.1155/2022/6952299

**Published:** 2022-09-16

**Authors:** Prashant S. Giri, Jahanvi Mistry, Mitesh Dwivedi

**Affiliations:** C. G. Bhakta Institute of Biotechnology, Faculty of Science, Uka Tarsadia University, Bardoli, Surat, 394 350 Gujarat, India

## Abstract

Vitiligo is a noncontagious autoimmune skin depigmenting disease. Regulatory T cells (Tregs) play a key role in maintaining peripheral tolerance; however, Tregs' number, suppressive function, and associated suppressive molecules (FOXP3, IL-10, and TGF-*β*) are found to be reduced in vitiligo patients. Although, the role of Tregs in vitiligo pathogenesis is well established, there are several contrary findings which suggest a controversial role of Tregs in vitiligo. Therefore, to clarify the role of Tregs in vitiligo pathogenesis, we aimed to study Tregs' frequency, suppressive capacity, and associated suppressive molecules (FOXP3, IL-10, and TGF-*β*) in vitiligo patients through meta-analysis approach. A total of 30 studies involving 1223 vitiligo patients and 1109 controls were included in the study. Pooled results from our meta-analysis suggested significantly reduced Treg cells' frequency in vitiligo patients (*p* = 0.002). Interestingly, Tregs' suppressive capacity was also significantly reduced in vitiligo patients (*p* = 0.0002); specifically, Treg-mediated suppression of CD8^+^T cells was impaired in vitiligo patients (*p* < 0.00001). Moreover, FOXP3, a key Tregs' transcription factor, was significantly reduced in blood and skin of vitiligo patients (*p* < 0.00001). Intriguingly, the FOXP3 expression was significantly reduced in the lesional skin as compared to perilesional and nonlesional skin (*p* = 0.007 and *p* = 0.04). Furthermore, the expression of key Treg-associated suppressive cytokines IL-10 and TGF-*β* were significantly reduced in vitiligo patients (*p* = 0.0005 and *p* = 0.01). The disease activity-based analysis suggested for reduced Tregs' frequency and FOXP3 expression in active vitiligo patients (*p* = 0.05 and *p* = 0.01). We also studied the effect of microRNA-based treatment, narrow band–UVB phototherapy, and Treg-associated treatments on Tregs' frequency, FOXP3, and IL-10 expression. Interestingly, we found increased Tregs' frequency, FOXP3, and IL-10 expression after the treatment (*p* = 0.007, *p* < 0.0001, and *p* = 0.002). Overall, our meta-analysis suggests that the Tregs play a crucial role in pathogenesis and progression of vitiligo, and hence, Treg-based therapeutic interventions could be effective in vitiligo patients.

## 1. Introduction

Vitiligo is a skin depigmenting disease, characterized by the loss of pigment-producing cells, melanocytes, resulting in the production of white scaly lesion on the skin's peripheral layer [1]. Its prevalence is about 0.5-2% worldwide [1, 2]. Currently, there is no effective treatment for vitiligo [3]. The pathophysiology of vitiligo is complicated and involves genetic, environment, oxidative stress, and autoimmunity factors [4–7]. Candidate gene studies highlight the role of autoimmunity in vitiligo, as polymorphisms in *IL1B*, *IL4*, *PSMB8*, *NLRP1*, *NP*Y, *FOXP3*, and *IFNG* genes were found to be associated with vitiligo [8–13].

Previous studies have shown the involvement of self-reactive CD8^+^ T cells, in the destruction of melanocytes [6, 14, 15]. Regulatory T cells (Tregs) control such autoimmune response [16]; however, vitiligo patients exhibited a reduced number of Treg cells [8, 14, 17–21] and decreased Treg cell suppressive function [21, 22]. Forkhead box P3 (FOXP3) is a key transcription factor of Tregs; it regulates the production of Tregs' suppressive molecules such as TGF-*β*, CTLA-4, IL-10, and GITR [23]. However, FOXP3 expression has been found to be altered in vitiligo patients [5, 9, 17–20, 22, 24–26]. Additionally, IL-10 and TGF-*β*, the key immunosuppressive cytokines produced by Tregs, govern the development of iTreg cells and participate in peripheral tolerance preservation and peripheral Treg cell maintenance [27]. However, IL-10 [5, 21, 22, 28–30] and TGF-*β* [5, 20–22, 28, 29, 31, 32] have also been reduced in vitiligo patients.

Although the previous studies strongly suggest the key role of Treg cells in vitiligo pathogenesis [8, 14, 17–21], the role of Tregs in vitiligo patients' peripheral immunological tolerance is still being contested, as few findings suggest increased or unaltered Treg cells' frequency [33–35], Tregs' suppressive function [14], FOXP3 [14, 33], and TGF-*β* [24, 36, 37] expression in vitiligo patients. Therefore, to overcome such contradiction, our current meta-analysis assessed the role of Treg cells in vitiligo pathogenesis by (i) investigating Treg cells' frequency in vitiligo patients; (ii) assessing Treg cells' suppressive capacity in vitiligo patients; (iii) determining FOXP3 expression levels in blood and skin of vitiligo patients; (iv) determining the expression levels of Tregs' suppressive cytokines: IL-10 and TGF-*β* in blood and skin of vitiligo patients; (v) carrying out disease activity-based analysis for Treg cells' frequency, FOXP3, IL-10, and TGF-*β* expression in vitiligo patients; and (vi) evaluating the effect of different therapeutic interventions on Treg cells' frequency, FOXP3, and IL-10 expression in vitiligo mice model and vitiligo patients through meta-analysis.

## 2. Materials and Methods

### 2.1. Literature Search

PubMed, Google Scholar, and Web of Science databases were searched up to 01st July 2022 for identifying studies evaluating the role of Tregs in vitiligo pathogenesis. The main keywords were “Tregs vitiligo,” “vitiligo,” “regulatory T cells,” “Treg,” “Treg cells number,” “Treg cells frequency,” “suppressive function,” “FOXP3,” “Forkhead box P3,” “IL-10,” “interleukin 10,” “transforming growth factor beta,” and “TGF-*β*.” The detailed search strategy is mentioned in Table S1. “The study protocol followed the Preferred Reporting Items for Systematic Reviews and Meta-Analyses (PRISMA) guidelines” (Table S2) [38, 39]. The studies were searched on the database for four different times independently by three investigators. All the reference lists in the relevant research papers were manually scanned.

### 2.2. Inclusion and Exclusion Criteria

The inclusion criteria included (1) original studies, (2) studies involving “vitiligo” and “regulatory T cells,” (3) studies assessing the Tregs' frequency in the skin or blood of vitiligo patients, (4) studies assessing the Tregs' suppressive capacity, and (5) studies assessing Tregs' suppressive molecules like FOXP3, IL-10, and TGF-*β*.

The criteria for exclusion were (1) review articles; (2) duplicate research; (3) studies that do not conduct quantitative assessments of Treg levels, Tregs' suppressive function, and Tregs' suppressive molecules like FOXP3, IL-10, and TGF-*β*; (4) meta-analysis; (5) studies that lack original data; and (6) studies with no full text.

### 2.3. Data Extraction

**Figure 1:**
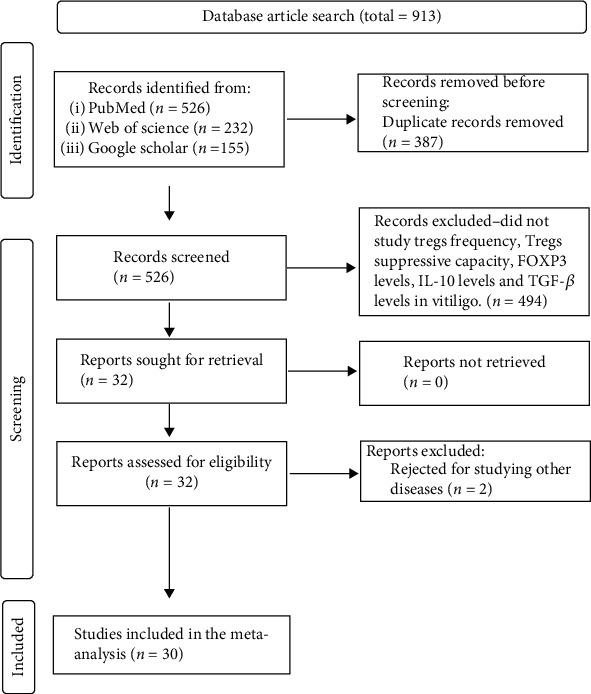
The flow chart of study selection for the meta-analysis. A total of 526 studies from PubMed, 232 studies from Web of Sciences, and 155 studies from Google Scholar databases were retrieved and processed for initial screening. After screening for the titles and abstracts, 387 duplicate records and 496 studies which did not study Tregs' frequency, Tregs' suppressive capacity, FOXP3 levels, IL-10 levels, and TGF-*β* levels in vitiligo were excluded. Finally, 30 studies consisting of total 1223 vitiligo patients and 1109 controls were included for the meta-analysis.

Figure 1 depicts the detailed screening methodology. Information such as author details, year of publication, sample size, Tregs' characterization, Tregs' frequency, Tregs' suppressive capacity, FOXP3 levels, IL-10 levels, TGF-*β* levels, information regarding the above-mentioned parameters being studied in the skin, blood, serum, or plasma of vitiligo patients, and disease activity are mentioned in Table 1. Patients with a persistent increase in lesions in the previous six months were classified as active vitiligo (AV) patients, while those without such progression were classified as stable vitiligo (SV) patients [5].

### 2.4. Quality Assessment of Enrolled Studies

The quality of the enrolled studies was assessed by three independent investigators. Initially, based on sample size, inclusion criteria, and methodology, the studies were screened. After the initial screening, the Newcastle-Ottawa Scale (NOS) criteria were employed to evaluate the quality of enrolled studies. “The NOS criteria were graded on a scale of 0 to 9, and three important criteria were included: (1) evaluation, (2) selection, and (3) ascertaining the outcome. Studies with a NOS score of five or higher were deemed as high-quality studies, while those with a lower score were deemed low-quality studies” [39].

### 2.5. Assessment of Publication Bias

“Publication bias in the enrolled studies was assessed by Eggers linear regression methods and test for publication bias using the JASP 0.14.1.0 software” [39, 40].

### 2.6. Sensitivity Analysis

To study the effect of individual studies on the meta-analysis, sensitivity analysis was carried out. The influence of the individual studies on the standardized mean difference (SMD) was evaluated before and after exclusion of each study.

### 2.7. Statistical Analysis

The meta-analysis provided quantitative data of standardized mean difference (SMD) as forest plots for Tregs' frequency, Tregs' suppressive capacity, FOXP3 levels, IL-10 levels, and TGF-*β* levels in vitiligo patients minus controls and for Tregs' frequency, Tregs' suppressive capacity, FOXP3 levels, and IL-10 levels in vitiligo patients and mice models posttreatment minus pretreatment. The random-effects model was utilized in the study as there were differences in experimental methods and techniques among selected studies. Meta-analysis was carried out using Review Manager 5.4 (Cochrane Collaboration, Oxford, United Kingdom). *p* ≤ 0.05 were considered statistically significant.

## 3. Results

### 3.1. Study Characteristics

913 results were collected from PubMed, Google Scholar, and Web of Science databases. After initial screening, 913 studies were excluded as they contained duplicate records and did not involve assessment of Tregs' frequency, Tregs' suppressive capacity, FOXP3 levels, IL-10 levels, and TGF-*β* levels in vitiligo. A total of 30 studies including 1223 vitiligo patients and 1109 controls were included in the meta-analysis. The study characteristics including author details, publication year, population size, Tregs' characterization, Tregs' frequency, Tregs' suppressive capacity, FOXP3 levels, IL-10 levels, TGF-*β* levels, information on the above-mentioned parameters investigated in the skin, blood, serum, or plasma of vitiligo patients, and disease activity are mentioned in Table 1. The studies included were in accordance with the quality assessment criteria, and the NOS score of 7 or 8 for the included studies suggests the high quality of these studies (Table S3).

### 3.2. Assessment of Publication Bias

The Eggers linear regression methods and test for publication bias suggested no significant publication bias for the meta-analysis (*p* = 0.594 and *p* = 0.319, respectively; Table S4), in vitiligo patients for the enrolled studies.

### 3.3. Treg Cells' Frequency in Vitiligo Patients

**Figure 2:**
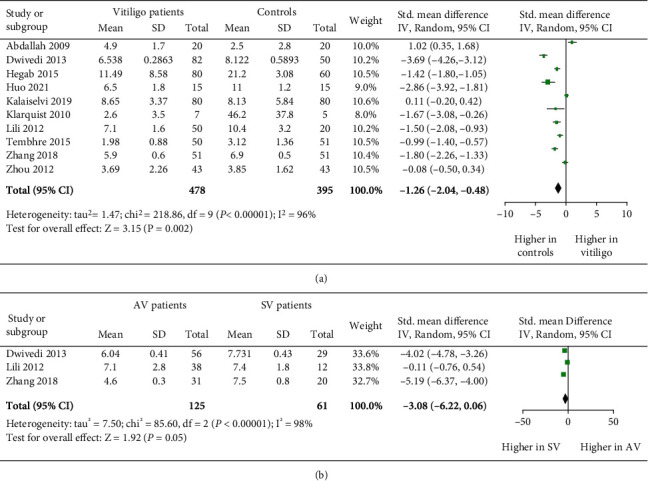
The forest plots for Treg cells' frequency in vitiligo patients and controls. (a) Treg cells' frequency in vitiligo patients vs. controls (*p* = 0.002; SMD: -1.26 [-2.04, -0.48]). (b) Treg cells' frequency in AV vs. SV patients (*p* = 0.05; SMD: -3.08[-6.22, 0.06]).

The proportion of Treg cells in vitiligo patients and controls were evaluated by calculating the standardized mean difference through meta-analysis. After initial screening, we found a total of 10 studies comprised of 478 vitiligo patients and 395 controls that assessed the Treg cells' frequency in vitiligo patients. Interestingly, we found significantly reduced Treg cells' frequency in vitiligo patients when compared to controls (*p* = 0.002, Figure 2(a)). The meta-analysis suggested that there was a 1.26 SMD decrease in Tregs' frequency in vitiligo patients (SMD: -1.26 [-2.04, -0.48], Figure 2(a)). Next, we carried out disease activity-based analysis for the frequency of Treg cells in vitiligo. However, only three studies comprised of 125 vitiligo patients and 61 controls carried out disease activity-based analysis for frequency of Treg cells. Our meta-analysis suggested a significant decrease in Treg cells' frequency in AV patients when compared to SV patients (*p* = 0.05, Figure 2(b)). Moreover, there was a difference of 3.08 SMD between AV patients and controls (SMD: -3.08 [-6.22, 0.06], Figure 2(b)). These findings from our meta-analysis suggested that the decrease in Treg cells numbers may lead to vitiligo pathogenesis and progression.

### 3.4. Treg Cells' Suppressive Capacity in Vitiligo Patients

**Figure 3:**
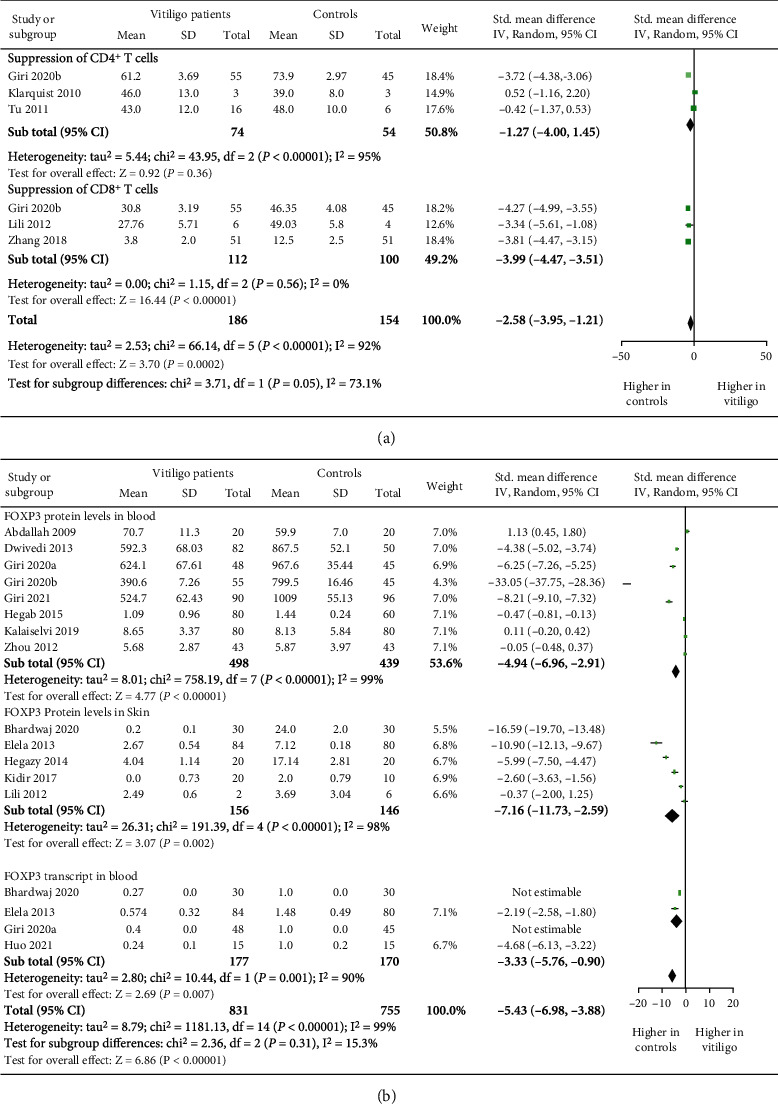
The forest plots for Tregs' suppressive capacity and FOXP3 expression in vitiligo patients and controls. (a) Tregs' suppressive capacity in vitiligo patients vs. controls (*p* = 0.0002; SMD: -2.58[-3.95, -1.21]). Treg-mediated suppression of CD4^+^ T cells in vitiligo patients vs. controls (*p* = 0.36; SMD: -1.27[-4.00, 1.45]). Treg-mediated suppression of CD8^+^ T cells in vitiligo patients vs. controls (*p* < 0.00001; SMD: -3.99[-4.47, -3.51]). (b) FOXP3 expression in skin and blood of vitiligo patients vs. controls (*p* < 0.00001; SMD: -5.43[-6.98, -3.88]). FOXP3 protein levels in blood of vitiligo patients vs. controls (*p* < 0.00001; SMD: -4.94 [-6.96, -2.91]). FOXP3 protein levels in skin of vitiligo patients vs. controls (*p* = 0.002, SMD: -7.16 [-11.73, -2.59]). *FOXP3* transcripts in blood of vitiligo patients vs. controls (*p* = 0.007, SMD: -3.33 [-5.76, -0.90]).

As the proportion of Treg cells in vitiligo patients was decreased, we studied the suppressive capacity of Tregs in vitiligo patients by calculating the standardized mean difference through meta-analysis. After screening we found that a total of 5 studies comprised of 186 vitiligo patients and 154 controls assessed the Treg cells' suppressive capacity in vitiligo. Interestingly, we found significant decrease in Treg cells' suppressive capacity between vitiligo patients and controls (*p* = 0.0002, Figure 3(a)). There was difference of 2.58 standardized mean between vitiligo patients and controls (SMD: -2.58 [-3.95, -1.21], Figure 3(a)). As there were 3 studies which accessed the Treg cells' suppressive capacity over CD4^+^ T cells and 3 studies accessed the Treg cells' suppressive capacity over CD8^+^ T cells, we carried out subgroup analysis to assess the Treg cells' suppressive capacity individually over CD4^+^ and CD8^+^ T cells. Our meta-analysis revealed significant decrease in Treg cells' suppressive capacity over CD8^+^ T cells in vitiligo patients (*p* < 0.00001, SMD: -3.99 [-4.47, -3.51], Figure 3(a)). However, there was no significant difference observed in Treg cells' suppressive capacity over CD4^+^ T cells in vitiligo patients when compared to controls (*p* = 0.36, Figure 3(a)). Although, the SMD of -1.27 suggests a trend of decreased Treg cells' suppressive capacity over CD4^+^ T cells in vitiligo patients (SMD: 1.27 [-4.00, 1.45], Figure 3(a)). Unfortunately, we could not carry out disease activity-based analysis for Treg cells' suppressive capacity in vitiligo, as only one of the five studies assessed the disease activity-based analysis for Treg cells' suppressive capacity in vitiligo patients. These results suggest for the crucial role of impaired Tregs' suppressive capacity in vitiligo pathogenesis.

### 3.5. FOXP3 Expression Levels in Blood and Skin of GV Patients

As FOXP3 is a key molecule for Treg cells' frequency and suppressive function, we evaluated the FOXP3 levels in GV patients and controls by calculating the standardized mean difference through meta-analysis. After the initial screening, we found that a total of 14 studies, comprised of 831 vitiligo patients and 755 controls, assessed the FOXP3 levels in vitiligo. Our meta-analysis suggested a significant decrease in FOXP3 levels in vitiligo patients when compared to controls (*p* < 0.00001, Figure 3(b)). The meta-analysis suggested that there was difference of 5.43 standardized mean for FOXP3 levels between vitiligo patients and controls (SMD: -5.43 [-6.98, -3.88]), Figure 3(b)). As the previous studies assessed the FOXP3 protein levels in blood, skin, and *FOXP3* transcript levels in blood, we also carried out subgroup analysis for the FOXP3 expression. Interestingly, our meta-analysis suggested significant decrease in FOXP3 protein levels in blood (*p* < 0.00001, SMD: -4.94 [-6.96, -2.91]), Figure 3(b)) and skin (*p* = 0.002, SMD: -7.16 [-11.73, -2.59]), Figure 3(b)) of vitiligo patients when compared to controls. Additionally, a significant decrease in *FOXP3* transcripts was observed in blood of vitiligo patients (*p* = 0.007, SMD: -3.33 [-5.76, -0.90]), Figure 3(b)). Next, we evaluated the FOXP3 expression in lesional, perilesional, and nonlesional skin. Interestingly, we found a significant decrease in FOXP3 levels in lesional skin when compared to perilesional skin (*p* = 0.007, SMD: -9.77 [-16.91, -2.62]) and nonlesional skin (*p* = 0.04, SMD: -1.01 [-1.95, -0.06]) in vitiligo patients (Figure S1a, b).

Further, we carried out disease activity-based analysis for FOXP3 expression in vitiligo. Previously, a total of four studies comprised of 179 vitiligo patients and 105 controls carried out disease activity-based analysis for FOXP3 expression. Our meta-analysis revealed significant decrease in FOXP3 protein expression in blood of AV patients when compared to SV patients (*p* = 0.01, Figure S1c). There was a difference of 2.99 SMD between AV and SV patients (SMD: -2.99 [-5.26, -0.71]), Figure S1c). These findings suggest the crucial role of FOXP3 in vitiligo pathogenesis and progression.

### 3.6. Expression of Treg-Associated Suppressive Cytokines (IL-10 and TGF-*β*) in Vitiligo

**Figure 4:**
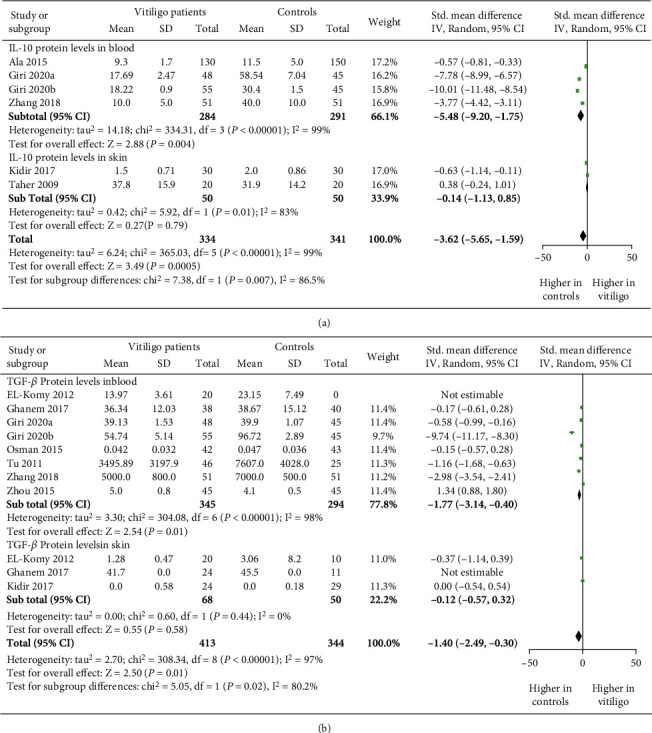
The forest plots for expression of Treg-associated suppressive cytokines (IL-10 and TGF-*β*) in vitiligo patients and controls. (a) IL-10 levels in blood and skin of vitiligo patients vs. controls (*p* = 0.0005; SMD: -3.62 [-5.65, -1.59]). IL-10 levels in blood of vitiligo patients vs. controls (*p* = 0.004; SMD: -5.48[-9.20, -1.75]). IL-10 expression levels in skin of vitiligo patients vs. controls (*p* = 0.79; SMD: -0.14 [-1.13, 0.85]). (b) TGF-*β* protein levels in blood and skin vitiligo patients vs. controls (*p* = 0.01, SMD: -1.40 [-2.49, -0.30]). TGF-*β* protein levels in blood of vitiligo patients vs. controls (*p* = 0.01, SMD: -1.77 [-3.14, -0.40]). TGF-*β* protein levels in skin of vitiligo patients vs. controls (*p* = 0.58, SMD: -0.12 [-0.57, 0.32]).

As our meta-analysis suggested impaired Tregs' suppressive capacity in vitiligo patients, we studied the expression levels of Treg-associated suppressive cytokines: IL-10 and TGF-*β* by calculating the standardized mean difference through meta-analysis. After the initial screening, we found that a total of 6 studies comprised of 334 vitiligo patients and 341 controls assessed the IL-10 levels in vitiligo. Interestingly, the meta-analysis revealed significant reduction in IL-10 protein levels in vitiligo patients when compared to controls (*p* = 0.0005, SMD: -3.62 [-5.65, -1.59]), Figure 4(a)). Further, to study the expression levels of IL-10 in blood and skin of vitiligo patients, we carried subgroup analysis. Interestingly, the meta-analysis showed significant decreased IL-10 protein levels in blood of vitiligo patients (*p* = 0.004, SMD: -5.48 [-9.20, -1.75] Figure 4(a)). However, there was no significant difference observed for IL-10 protein levels in skin of vitiligo patients (p = 0.79, SMD: -0.14 [-1.13, 0.85], Figure 4(a)). Next, to study the role of IL-10 on disease activity, we carried out disease activity-based analysis for IL-10 levels in vitiligo. However, we found only three studies comprised of 154 vitiligo patients and 79 controls and carried out the disease activity-based analysis for IL-10 expression. Our meta-analysis suggested that there was no significant decrease in IL-10 protein expression in AV patients when compared to SV patients (*p* = 0.09, SMD: -1.67 [-3.59, 0.25]) Figure S2a). Although, the SMD of -1.67 suggests for a trend of decreased TGF-*β* levels in AV patients (SMD: -1.67 [-3.59, 0.25]), Figure S2a).However, the SMD of -1.67 between AV and SV patients, suggested the trend for decreased TGF-*β* levels in AV patients (SMD: -1.67 [-3.59, 0.25]), Figure S2a). These findings suggest for the crucial role of IL-10 in vitiligo pathogenesis.

Furthermore, we studied the expression of TGF-*β* by calculating the standardized mean difference through meta-analysis in vitiligo patients and controls. After the initial screening, we found that a total of 8 studies comprised of 389 vitiligo patients and 333 controls assessed TGF-*β* levels in vitiligo. Our meta-analysis revealed a significant decrease in TGF-*β* protein levels in vitiligo patients when compared to controls (*p* = 0.01, SMD: -1.40 [-2.49, -0.30]), Figure 4(b)). In addition, we studied the expression of TGF-*β* in blood and skin of vitiligo patients by subgroup analysis. Interestingly, we found significant decrease in TGF-*β* protein levels in blood of vitiligo patients (*p* = 0.01, SMD: -1.77 [-3.14, -0.40]), Figure 4(b)). However, no significant difference was observed for TGF-*β* protein levels in skin of vitiligo patients (*p* = 0.58, SMD: -0.12 [-0.57, 0.32]), Figure 4(b)). Further, we carried out disease activity-based analysis to study the role of TGF-*β* on disease activity. However, only two studies comprised of 71 vitiligo patients and 32 controls carried out disease activity-based analysis for the TGF-*β* expression. There was no significant decrease observed in TGF-*β* protein expression in AV patients when compared to SV patients (*p* = 0.06, Figure S2b). Although, the SMD of -3.49 suggests for a trend of decreased TGF-*β* levels in AV patients (SMD: -3.49 [-7.08, 0.10]), Figure S2b). These findings suggest for the crucial role of TGF-*β* in GV pathogenesis.

### 3.7. Effect of Different Treatment on Tregs' Frequency, FOXP3, and IL-10 Levels in Vitiligo

**Figure 5:**
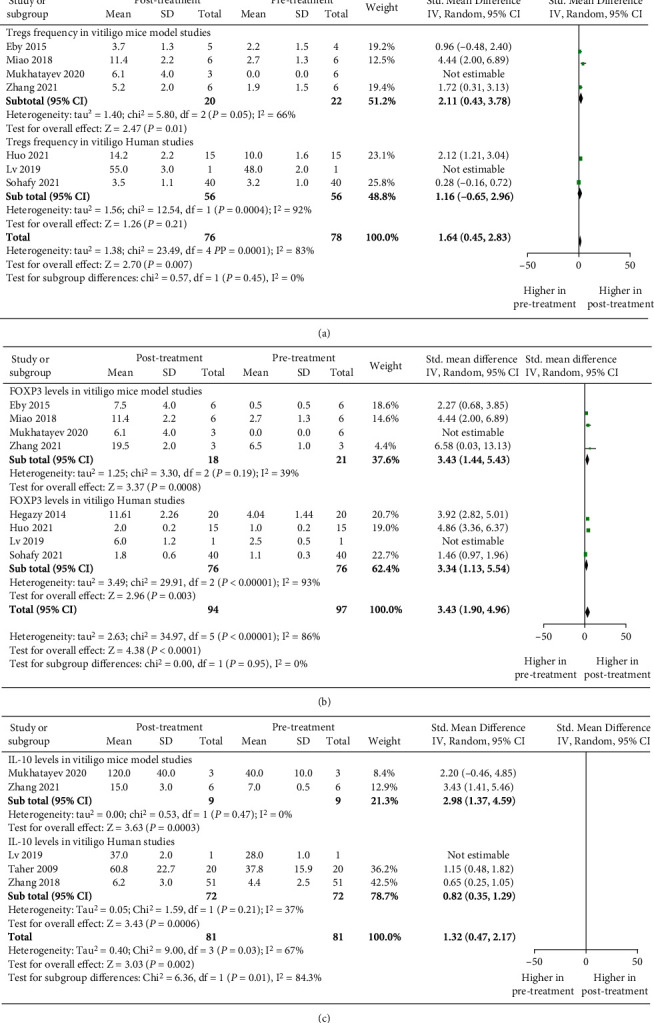
The forest plots for Tregs' frequency, FOXP3, and IL-10 levels in vitiligo pre- and posttreatment. (a) Tregs' frequency posttreatment vs. pretreatment (*p* = 0.007, SMD: 1.64 [0.45, 2.83]). Tregs' frequency posttreatment vs. pretreatment in mouse models of vitiligo (*p* = 0.01, SMD: 2.11 [0.43, 3.78]). Tregs' frequency post treatment vs. pretreatment in human studies for vitiligo (*p* = 0.21 SMD: 1.16 [-0.65, 2.96]). (b) FOXP3 protein levels posttreatment vs. pretreatment (*p* < 0.0001, SMD: 3.43 [1.90, 4.96]). FOXP3 protein levels posttreatment vs. pretreatment in vitiligo mice model study (*p* = 0.0008, SMD: 3.43 [1.44, 5.43]). FOXP3 protein levels posttreatment vs. pretreatment in human studies (*p* = 0.003, 3.34 [1.13, 5.54]). (c) IL-10 levels posttreatment vs. pretreatment in vitiligo patients (*p* = 0.002, SMD: 1.32 [0.47, 2.17]). IL-10 levels posttreatments vs. pretreatment in vitiligo mice models study (*p* = 0.0003, SMD: 2.98 [1.37, 4.59]). IL-10 levels post treatment vs. pretreatment in vitiligo human studies (*p* = 0.0006, 0.82 [0.35, 1.29]).

To study the impact of microRNA-based treatment, narrow band–UVB phototherapy, and Treg-associated treatments on Tregs' frequency, FOXP3, and IL-10 levels in vitiligo, we assessed the Tregs' frequency, FOXP3, and IL-10 levels in vitiligo patients and mouse model of vitiligo, pre- and posttreatment by calculating the standardized mean difference through meta-analysis. There were a total of 7 studies comprised of 56 vitiligo patients (human studies) and 22 vitiligo mice (animal studies) that studied the Tregs' frequency, pre- and posttreatment. Interestingly, the meta-analysis revealed significant increase in Treg cells' frequency after the treatment (*p* = 0.007, SMD: 1.64 [0.45, 2.83]), Figure 5(a)). Furthermore, the subgroup analysis suggested significant increase in Treg cells' frequency after the treatment in mouse models of vitiligo (*p* = 0.01, 2.11 [0.43, 3.78]), Figure 5(a)). However, there was no significant difference found in Treg cells' frequency after the treatment, in human studies (*p* = 0.21, Figure 5(a)). However, the SMD of 1.16 between treatment groups, suggested the trend for increased Treg cells' frequency after treatment (SMD: 1.16 [-0.65, 2.96]), Figure 5(a)). Although, the SMD of 1.16 suggests a trend of increased Treg cells' frequency after the treatment (SMD: 1.16 [-0.65, 2.96]) Figure 5(a)).

**Figure 6:**
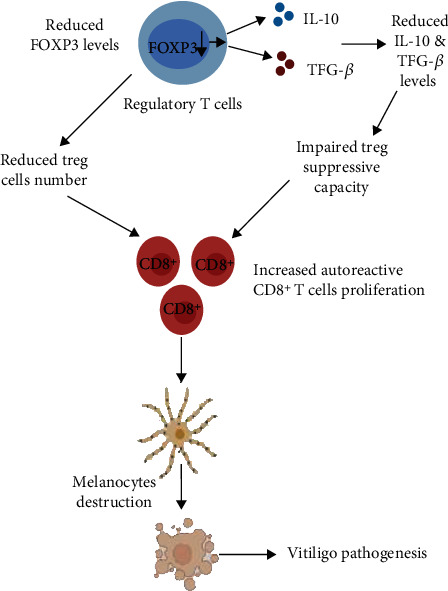
Role of regulatory T cells in vitiligo pathogenesis. The reduced expression of FOXP3, the key Tregs' transcription factors of Tregs, results in decreased expression of Tregs' suppressive cytokines (IL-10 and TGF-*β*) and impaired Tregs' suppressive capacity. Moreover, the reduced FOXP3 levels in the blood suggests reduced Tregs' number in vitiligo patients. Thus, the decreased Tregs' frequency and impaired Tregs' suppressive capacity lead to unchecked CD8^+^ T proliferation, which results into melanocyte death and vitiligo pathogenesis.

Further, we evaluated FOXP3 expression posttreatment. A total of 8 studies comprised of 76 vitiligo patients (human studies) and 21 vitiligo mice (animal studies) studied the FOXP3 protein expression pre- and posttreatment. Our meta-analysis indicated a significant increase in FOXP3 protein expression after the treatment (*p* < 0.0001, SMD: 3.43 [1.90, 4.96]. Moreover, subgroup analysis revealed significant increase in FOXP3 expression after the treatment, in human studies (*p* = 0.003, 3.34 [1.13, 5.54]), Figure 5(b)) and vitiligo mice model studies (*p* = 0.0008, SMD: 3.43 [1.44, 5.43]), Figure 5(b)). Additionally, we assessed IL-10 expression posttreatment. A total of 5 studies comprised of 72 vitiligo patients (human studies) and 9 vitiligo mice (animal studies) studied the IL-10 protein levels pre- and posttreatment. Our meta-analysis revealed a significant increase in IL-10 protein levels after the treatment (*p* = 0.002, SMD: 1.32 [0.47, 2.17]), Figure 6(c)). Moreover, the subgroup analysis suggested significant increase in IL-10 protein levels after the treatment, in human studies (*p* = 0.0006, 0.82 [0.35, 1.29]), Figure 5(c)) and vitiligo mice model studies (*p* = 0.0003, SMD: 2.98 [1.37, 4.59]), Figure 5(c)).

### 3.8. Sensitivity Analysis

We carried out sensitivity analysis to assess the influence of individual studies on the overall SMD. There were no outlying studies found to influence a significant change in the SMD (Table S5-S12).

## 4. Discussion

Treg cells maintain peripheral immune tolerance by actively suppressing self-reactive T cells [41]. Functional alteration in Treg cells lead to various autoimmune diseases [42]. Similarly, Treg cells have a critical role in vitiligo pathogenesis [5, 8, 14, 17–22, 24–26]. However, few contrary findings [14, 33–35] suggest a controversial role of Tregs in vitiligo pathogenesis. Therefore, we performed a meta-analysis to study Treg cells' frequency, Tregs' suppressive function, FOXP3, IL-10, and TGF-*β* expression in vitiligo patients. The pooled results of our meta-analysis suggested a significant decrease in Tregs' frequency in vitiligo patients (Figure 2(a)). Particularly, there was 1.26 SMD decrease in Treg cells' frequency in vitiligo patients (Figure 2(a)). Our results are strongly supported by previous studies suggesting significantly decreased Treg cells levels in vitiligo patients [8, 14, 17–21]. However, they contrast with few reports [33–35]. The conflicting results may be due to differences in sample size, difference in methodology used, and ethnicity differences. Additionally, difference in antibody clones used for flow cytometry studies could also account for differences in Tregs' number [8]. Moreover, the differences in the characterization of Tregs may also be responsible for the conflicting results [43], suggesting that future studies should use strict and consistent markers for Treg cell characterization in vitiligo. Interestingly, the disease activity-based analysis suggested the role of reduced Treg cells in vitiligo disease activity, which is in concordance to the previous studies [8, 18, 21]. Overall, our meta-analysis taking into consideration all the available data suggested that the decreased Treg cells' frequency might be involved in vitiligo pathogenesis and progression.

As we found decreased Tregs' frequency in vitiligo patients, it was pertinent to assess Tregs' suppressive capacity in vitiligo patients. Interestingly, our meta-analysis suggested significantly decreased Treg cells' suppressive capacity in vitiligo patients (Figure 3(a)). The results agreed with that of the previous studies [18, 21, 22]. Further, the subgroup analysis revealed a significant decrease in Treg-mediated suppression of CD8^+^ T cells' proliferation in vitiligo patients (Figure 3(a)). These findings were in concordance with the previous studies [18, 21, 22]. The autoreactive CD8^+^ T cells are the major culprits responsible for the destruction of melanocytes in vitiligo patients [6, 14, 15]. Our meta-analysis together with the previous studies suggest for the crucial role of Tregs and CD8^+^ T cells in vitiligo pathogenesis [6, 14, 15]. Surprisingly, we did not find significant difference in Treg-mediated suppression of CD4^+^ T cells in vitiligo patients (Figure 3(a)). This might be due to lower sample size in the available studies, difference in detection of effector cells proliferation, i.e., BrdU ELISA or [^3^H] thymidine incorporation assay, and differences in activation of T cells, i.e., through antigen presenting cells or CD3/CD28 beads; hence, further studies with larger sample size and uniform detection methods are needed to confirm these findings. However, our meta-analysis suggested a trend for decreased Treg-mediated suppression of CD4^+^ T cells in vitiligo patients as there was a 1.27 SMD decreased in Treg-mediated suppression of CD4^+^ T cells in vitiligo patients (Figure 3(a)). Nevertheless, our meta-analysis suggested the crucial role of impaired Tregs' suppressive capacity in vitiligo pathogenesis.

As FOXP3 is an indispensable molecule for Tregs' number and suppressive function [44], next we assessed FOXP3 expression levels in blood and skin of vitiligo patients. Our meta-analysis suggested significantly decreased FOXP3 expression levels in blood and skin of vitiligo patients (Figure 3(b)). Additionally, our meta-analysis suggested the role of decreased FOXP3 expression in disease activity of vitiligo (Figure S1a). These results were in concordance with the previous studies [5, 9, 17–20, 22, 24–26]. The decreased FOXP3 protein and transcript levels in the blood may be due to the reduced Tregs' frequency in vitiligo patients; therefore, our meta-analysis further suggests for the role of decreased Tregs' frequency in vitiligo pathogenesis. Moreover, the FOXP3 expression was significantly reduced in lesional skin as compared to perilesional and nonlesional skin of vitiligo patients (Figure S1b, c), indicating that FOXP3 expressing Treg cells are indeed crucial for suppressing autoreactive melanocyte-specific CD8^+^ T cells in lesional skin. Since FOXP3 plays a crucial role in phenotype and suppressive function of Tregs, our recent study also reported a positive correlation of FOXP3 levels with Treg cells' suppressive capacity in vitiligo [22]. Therefore, the decreased expression of FOXP3 in lesional skin, as suggested by our meta-analysis, further establishes the role of impaired Tregs' suppressive capacity in vitiligo pathogenesis.

Furthermore, FOXP3 governs the expression of downstream Tregs' suppressive cytokines such as IL-10 and TGF-*β* [23]. Apart from cell-to-cell contact, Treg cells maintain peripheral tolerance by secreting immunosuppressive cytokines such as IL-10 and TGF-*β* [45]. Moreover, IL-10 in presence of TGF-*β* also improves Treg cell expansion [45]. Hence, we further evaluated the role of IL-10 and TGF-*β* in vitiligo. Interestingly, our meta-analysis suggested a significant decrease in IL-10 expression levels in vitiligo patients more specifically in blood of vitiligo patients (Figure 4(a)). These findings were in concordance with the previous studies [5, 21, 22, 28–30] indicating the indispensable role of IL-10 in vitiligo pathogenesis. TGF-*β* is a pleotropic immunosuppressive cytokine, which regulates the immune response by suppressing T and B cells. Additionally, it plays a crucial role in proliferation and induction of Tregs [46]. Interestingly, the meta-analysis suggested significant decrease in TGF-*β* levels in vitiligo patients (Figure 4(b)). These results corroborate with the previous studies [5, 20–22, 28, 29, 31, 32]; however, these results contrast with few reports [24, 36, 37]. The contrasting results may be due to differences in sample size and methodology used for TGF-*β* detection, i.e., differences in ELISA kits with varying sensitivities and differences in antibodies used for detection of TGF-*β*. Moreover, there was no difference in IL-10 and TGF-*β* expression levels in skin of vitiligo patients, which might be due to lower sample size and differences in IL-10 and TGF-*β* detection techniques, i.e., ELISA and immunohistochemical staining. Furthermore, we could only find two studies assessing IL-10 and TGF-*β* expression levels in skin of vitiligo patients; hence, future studies with larger sample size are needed to confirm these findings. Additionally, we could not find significant difference in IL-10 and TGF-*β* levels between AV and SV patients (Figure S2a, b), which could be due to the less sample size and lower number of studies assessing disease activity-based analysis. However, we observed a trend of decreased IL-10 and TGF-*β* levels in AV patients, as there was a 1.67 and 3.49 SMD decrease in IL-10 and TGF-*β* levels, respectively (Figure S2a, b), indicating a likely role of IL-10 and TGF-*β* in vitiligo progression. Overall, our meta-analysis with the previously available studies suggests the crucial role of IL-10 and TGF-*β* in vitiligo pathogenesis.

Interestingly, our recent study has also suggested that the decreased expression of FOXP3 leads to impaired Tregs' suppressive capacity in vitiligo [22]. Therefore, our meta-analysis taking into consideration all the available data suggests that the reduced FOXP3 expression levels could lead to decreased downstream Tregs' suppressive cytokines IL-10 and TGF-*β*, thereby results into impaired Tregs' suppressive capacity. Moreover, the decreased Tregs' frequency and impaired Tregs' suppressive function could lead to widespread activation and expansion of CD8^+^ T cells resulting into melanocytes destruction and vitiligo pathogenesis (Figure 6).

Furthermore, we evaluated Tregs' frequency, FOXP3, and IL-10 expression posttreatment in vitiligo. Interestingly, out meta-analysis revealed increased Treg cells' frequency, FOXP3, and IL-10 expression after microRNA-based treatment, narrow band–UVB phototherapy, and Treg-associated treatments in vitiligo (Figures 5(a)–5(c)). The subgroup analysis suggested significant increase in Tregs' frequency, FOXP3, and IL-10 levels in vitiligo mice model studies (Figures 5(a)–5(c)). Additionally, we found significantly, increased FOXP3 and IL-10 levels in human studies (Figures 5(b) and 5(c)). However, we did not find significant increase in Tregs' frequency posttreatment in human studies (Figure 5(a)), which could be due to lower number of studies and less sample size. Nevertheless, our meta-analysis suggested a trend of increased Treg cells' frequency after treatment as there was 1.16 SMD increase in Tregs' frequency after treatment (Figure 5(a)). Moreover, our meta-analysis suggested improved Tregs' frequency, FOXP3, and IL-10 expression posttreatment in human and mice model studies, which is in concordance to the previous studies [47–53]. According to the studies included in the meta-analysis, treating vitiligo mouse models with antigen-specific CAR Tregs, PD-L1 fusion peptides, and CCL22 DNA reverses depigmentation by increasing the number of Tregs and FOXP3 expression in the skin [47, 50, 51]. Additionally, polymeric nanoparticles containing rapamycin and autoantigens induce antigen-specific immune tolerance, thereby inhibiting vitiligo in mouse models of vitiligo [53]. Furthermore, in human studies, tacrolimus, miR-155, and HO-1 increase the IL-10 expression in vitiligo lesions, whereas narrow band-UVB phototherapy, miR-21-5p, and miR-155 enhance the Tregs' frequency and FOXP3 levels, thereby suppressing the melanocyte destruction caused by unregulated Th1 pathways [19, 21, 48, 49, 52]. Therefore, such Treg-based therapeutics can control depigmentation and support immune tolerance in vitiligo. Additionally, our recent study has highlighted that calcium treatment significantly increases Treg cells' suppressive capacity in vitiligo pathogenesis [54]. Overall, our meta-analysis taking into consideration all the available data suggests that targeting Treg cells in vitiligo patients could lead to effective Treg-based therapeutics for vitiligo.

The limitations of the meta-analysis were inclusion of only the studies published in English language, interstudy heterogeneity, and analysis based on the type of vitiligo which could not be carried out due to scarcity of such studies involving type of vitiligo-based analysis. Moreover, the role of innate lymphoid cells subpopulations (ILC1, ILC2, ILC3, and ILCregs) in pathogenesis of various autoimmune and inflammatory diseases including Crohn's disease, atopic dermatitis, inflammatory bowel disease, psoriasis, multiple sclerosis, and colitis, has been suggested [55]. However, studies assessing the role of ILC in vitiligo are lacking; hence, it was not included in this meta-analysis. Nevertheless, the strengths of our meta-analysis were no publication bias for the enrolled studies and high statistical power due to population diversity. Additionally, sensitivity analysis suggested no influence of single study on overall SMD. Moreover, FOXP3, IL-10, and TGF-*β* levels in blood and skin were assessed by subgroup analysis, and disease activity-based analysis was also carried out.

## 5. Conclusions

This is the first meta-analysis conducted for confirming the role of Tregs in vitiligo pathogenesis. The pooled results of the meta-analysis suggested for crucial role of decreased Treg cells' frequency and FOXP3 expression in vitiligo pathogenesis and progression. The meta-analysis suggested an impaired Tregs' suppressive capacity in vitiligo patients; particularly the meta-analysis highlighted the reduced Treg-mediated suppression of CD8^+^ T cells in vitiligo patients, which was also supported by decreased levels of key immunosuppressive cytokines (IL-10 and TGF-*β*). However, more number of studies is warranted with larger sample size for confirming the role of Tregs in vitiligo progression. This meta-analysis also revealed an increase in Tregs' frequency, FOXP3 expression, and IL-10 expression in vitiligo after microRNA-based treatment, narrow band–UVB phototherapy, and Treg-associated treatments, indicating that targeting Treg cells in vitiligo patients could lead to effective therapeutics.

## Figures and Tables

**Table 1:** Study characteristics.

Study	Year	Vitiligo patients/controls	Skin/blood	Treg characterization	Tregs' frequency	Tregs' suppressive capacity	FOXP3 levels	IL-10 levels	TGF-*β* levels	Disease activity-based analysis
Abdallah et al. [33]	2009	20/20	Blood	CD4^+^CD25high FoxP3^+^	Increased	NA	Increased	NA	NA	No
Ala et al.[30]	2015	130/150	Blood	NA	NA	NA	NA	Decreased	NA	No
Bhardwaj et al.[24]	2020	30/30	Skin	NA	NA	NA	Decreased	NA	Increased	No
Dwivedi et al.[8]	2013	82/50	Blood	CD4^+^CD25hiFOXP3^+^	Decreased	NA	Decreased	NA	NA	No
Eby et al.[50]	2015	7/6	Skin	CD3^+^FOXP3^+^	Increased after treatment	NA	NA	NA	NA	No
Abou Elela et al.[25]	2013	84/80	Blood	CD4^+^ CD25^+^ FOXP3^+^	NA	NA	Decreased	NA	NA	Yes (VIDA)
El-Komy et al.[28]	2012	20/10	Skin	NA	NA	NA	NA	NA	Decreased	Yes (VIDA)
Ghanem et al.[36]	2017	38/40	Serum/skin	NA	NA	NA	NA	NA	No difference	No
Giri et al.[5]	2020	55/45	Blood	CD3^+^CD25^+^ T cells	NA	NA	Decreased	Decreased	Decreased	Yes
Giri et al.[22]	2020	48/45	Blood	CD3^+^CD25^+^ T cells	NA	Decreased	Decreased	Decreased	Decreased	Yes
Giri et al.[9]	2021	96/90	Blood	CD3^+^CD25^+^ T cells	NA	NA	Decreased	NA	NA	Yes
Hegab et al.[17]	2015	80/60	Blood	CD4^+^CD25^+^	Decreased	NA	Decreased	NA	NA	Yes (VIDA)
Hegazy et al.[26]	2014	20/20	Skin	NA	NA	NA	Decreased	NA	NA	Yes (VIDA)
Huo et al.[19]	2021	15/15	Blood	CD4^+^CD25^+^CD127^−^	Decreased	NA	Decreased	NA	NA	No
Kalaiselvi et al.[34]	2019	80/80	Blood	CD4^+^FOXP3^+^	No difference	NA	NA	NA	NA	Yes (VIDA)
Kidir et al.[29]	2017	30/30	Skin	NA	NA	NA	NA	Decreased	Decreased	No
Klarquist et al.[14]	2010	07/05	Skin	CD4^+^CD25^+^CD127^−^FOXP3^+^	Decreased	No difference	Increased	NA	NA	No
Lili et al.[18]	2012	50/20	Blood	CD4^+^CD25^+^CD127^−^	Decreased	Decreased	Decreased	NA	NA	No
Lv et al.[49]	2019	1/1	Blood/skin	CD4^+^ CD25^+^ FOXP3^+^	Increased after treatment	NA	Increased after treatment	Increased after treatment	Increased after treatment	No
Miao et al.[51]	2018	8/9	Skin	FOXP3^+^CD3^+^	Increased after treatment	NA	NA	NA	NA	No
Mukhatayev et al.[47]	2020	11/12	Skin	CD4^+^ FOXP3^+^	Increased after treatment	NA	NA	Increased after treatment	NA	No
Osman et al.[31]	2015	42/43	Blood	NA	NA	NA	NA	NA	Decreased	No
Sohafy et al.[48]	2021	40/40	Blood	CD4^+^ CD25^+^FOXP3^+^	Increased after treatment	NA	NA	NA	NA	No
Taher et al.[52]	2009	20/20	Skin	NA	NA	NA	NA	Increased after treatment	NA	No
Tembhre et al.[20]	2015	50/51	Skin	NA	Decreased	NA	Decreased	NA	Decreased	No
Tu et al.[32]	2017	46/25	Serum	CD4^+^ CD25^+^	NA	No difference	NA	NA	Decreased	No
Zhang et al.[21]	2018	51/51	Blood	CD4^+^CD25^high^	Decreased	Decreased	NA	Decreased	Decreased	No
Zhang et al.[53]	2021	3/3	Blood/skin	FOXP3^+^ CD4^+^	NA	NA	NA	Increased after treatment	NA	No
Zhou et al.[35]	2012	43/43	Blood	CD4^+^ CD25^+^ FOX3^+^	No difference	NA	NA	NA	NA	No
Zhou et al.[37]	2015	45/45	Serum	NA	NA	NA	NA	NA	Increased	No

Abbreviations: NA: not applicable; VIDA: vitiligo disease activity score; Treg: regulatory T cells; IL-10: interleukin 10; TGF-*β*: transforming growth factor beta.

## Data Availability

Data will be available on request from the authors.

## Supplementary Materials

Supplementary 1 Table S1: search strategy for inclusion of studies in the meta-analysis. Table S2: PRISMA checklist. Table S3: Newcastle–Ottawa quality assessment scale for selected studies. Table S4: test for publication bias. Table S5: sensitivity analysis: standardized mean difference for Tregs' frequency in vitiligo patients and controls. Table S6: sensitivity analysis: standardized mean difference for Tregs' suppressive capacity levels in vitiligo patients and controls. Table S7: sensitivity analysis: standardized mean difference for FOXP3 protein levels in vitiligo patients and controls. Table S8: sensitivity analysis: standardized mean difference for IL-10 protein levels in vitiligo patients and controls. Table S9: sensitivity analysis: standardized mean difference for TGF-*β* levels in vitiligo patients and controls. Table S10: sensitivity analysis: standardized mean difference for Treg levels in vitiligo posttreatment. Table S11: sensitivity analysis: standardized mean difference foxp3 levels in vitiligo post treatment. Table S12: sensitivity analysis: standardized mean difference for IL-10 levels in vitiligo post treatment.

Supplementary 2 Figure S1: the forest plots for FOXP3 expression levels in active vitiligo and stable vitiligo patients and lesional, perilesional, and nonlesional skin of vitiligo patients. (a) FOXP3 protein expression in active vitiligo patients vs. stable vitiligo patients (*p* = 0.01; SMD: -2.99 [-5.26, -0.71]). (b) FOXP3 levels in lesional skin vs. perilesional skin (*p* = 0.007, SMD: -9.77 [-16.91, -2.62]). (c) FOXP3 levels in lesional skin vs. nonlesional skin (*p* = 0.04, SMD: -1.01 [-1.95, -0.06]). Figure S2: the forest plots for IL-10 and TGF-*β* expression levels in active vitiligo and stable vitiligo patients. (a) IL-10 protein expression in active vitiligo patient's vs. stable vitiligo patients (*p* = 0.09, SMD: -1.67 [-3.59, 0.25]). (b) TGF-*β* levels in active vitiligo patient's vs. stable vitiligo patients (*p* = 0.06; SMD: -3.49 [-7.08, 0.10]).
