# Supplementary material for: Meta-Analysis of Alterations in Regulatory T Cells' Frequency and Suppressive Capacity in Patients with Vitiligo
Source: J Immunol Res. 2022 Sep 16;2022:6952299. doi: 10.1155/2022/6952299 (PMC9508461; doi:10.1155/2022/6952299)
Supplement: Supplementary 1 — Table S1: search strategy for inclusion of studies in the meta-analysis. Table S2: PRISMA checklist. Table S3: Newcastle–Ottawa quality assessment scale for selected studies. Table S4: test for publication bias. Table S5: sensitivity analysis: standardized mean difference for Tregs' frequency in vitiligo patients and controls. Table S6: sensitivity analysis: standardized mean difference for Tregs' suppressive capacity levels in vitiligo patients and controls. Table S7: sensitivity analysis: standardized mean difference for FOXP3 protein levels in vitiligo patients and controls. Table S8: sensitivity analysis: standardized mean difference for IL-10 protein levels in vitiligo patients and controls. Table S9: sensitivity analysis: standardized mean difference for TGF-β levels in vitiligo patients and controls. Table S10: sensitivity analysis: standardized mean difference for Treg levels in vitiligo posttreatment. Table S11: sensitivity analysis: standardized mean difference foxp3 levels in vitiligo post treatment. Table S12: sensitivity analysis: standardized mean difference for IL-10 levels in vitiligo post treatment. [file 6952299.f1.doc]

| **Table S1. Search strategy for inclusion of studies in the meta-analysis.** | | | |
| --- | --- | --- | --- |
| **Sr. No.** | **Database** | **Query** | **Results** |
| **1** | PubMed | “Tregs vitiligo” AND "vitiligo” OR "regulatory T cells” OR "Treg" OR “suppressive function” OR “FOXP3” OR “forkhead box P3” OR “IL-10”, “interleukin 10” OR “transforming growth factor beta” OR “TGF-β“ | 556 |
| **2** | Web of science | “Tregs vitiligo” AND "vitiligo” OR "regulatory T cells” OR "Treg" OR “suppressive function” OR “FOXP3” OR “forkhead box P3” OR “IL-10”, “interleukin 10” OR “transforming growth factor beta” OR “TGF-β“ | 232 |
| 3 | Google Scholar | “Tregs vitiligo” AND "vitiligo” OR "regulatory T cells” OR "Treg" OR “suppressive function” OR “FOXP3” OR “forkhead box P3” OR “IL-10”, “interleukin 10” OR “transforming growth factor beta” OR “TGF-β“ | 155 |

| **Table S2: Prisma checklist** | | | |
| --- | --- | --- | --- |
| **Section and Topic** | **Item #** | **Checklist item** | **Location where item is reported** |
| **TITLE** | | |  |
| Title | 1 | Identify the report as a systematic review. | Title  Page No. 1 |
| **ABSTRACT** | | |  |
| Abstract | 2 | See the PRISMA 2020 for Abstracts checklist. | Abstract  Page No. 2 |
| **INTRODUCTION** | | |  |
| Rationale | 3 | Describe the rationale for the review in the context of existing knowledge. | Introduction  Page No. 3,4 |
| Objectives | 4 | Provide an explicit statement of the objective(s) or question(s) the review addresses. | Introduction  Page No. 3,4 |
| **METHODS** | | |  |
| Eligibility criteria | 5 | Specify the inclusion and exclusion criteria for the review and how studies were grouped for the syntheses. | Materials and methods: Inclusion and exclusion criteria  Page No. 4 |
| Information sources | 6 | Specify all databases, registers, websites, organisations, reference lists and other sources searched or consulted to identify studies. Specify the date when each source was last searched or consulted. | Materials and methods: Literature search  Page No. 4 |
| Search strategy | 7 | Present the full search strategies for all databases, registers and websites, including any filters and limits used. | Table S1 |
| Selection process | 8 | Specify the methods used to decide whether a study met the inclusion criteria of the review, including how many reviewers screened each record and each report retrieved, whether they worked independently, and if applicable, details of automation tools used in the process. | Materials and methods: Inclusion and exclusion criteria  Page No. 4 |
| Data collection process | 9 | Specify the methods used to collect data from reports, including how many reviewers collected data from each report, whether they worked independently, any processes for obtaining or confirming data from study investigators, and if applicable, details of automation tools used in the process. | Materials and methods: Data extraction  Page No. 4 |
| Data items | 10a | List and define all outcomes for which data were sought. Specify whether all results that were compatible with each outcome domain in each study were sought (e.g. for all measures, time points, analyses), and if not, the methods used to decide which results to collect. | Results  Study characteristics Table 1 |
|  | 10b | List and define all other variables for which data were sought (e.g. participant and intervention characteristics, funding sources). Describe any assumptions made about any missing or unclear information. | Results  Study characteristics Table 1 |
| Study risk of bias assessment | 11 | Specify the methods used to assess risk of bias in the included studies, including details of the tool(s) used, how many reviewers assessed each study and whether they worked independently, and if applicable, details of automation tools used in the process. | Materials and methods: Assessment of publication bias  Page No.4 |
| Effect measures | 12 | Specify for each outcome the effect measure(s) (e.g. risk ratio, mean difference) used in the synthesis or presentation of results. | Materials and methods: Statistical analysis  Page No.4 |
| Synthesis methods | 13a | Describe the processes used to decide which studies were eligible for each synthesis (e.g. tabulating the study intervention characteristics and comparing against the planned groups for each synthesis (item #5)). | Results  Study characteristics Table 1 |
|  | 13b | Describe any methods required to prepare the data for presentation or synthesis, such as handling of missing summary statistics, or data conversions. |  |
|  | 13c | Describe any methods used to tabulate or visually display results of individual studies and syntheses. | Materials and methods: Statistical analysis  Page No.4 |
|  | 13d | Describe any methods used to synthesize results and provide a rationale for the choice(s). If meta-analysis was performed, describe the model(s), method(s) to identify the presence and extent of statistical heterogeneity, and software package(s) used. | Materials and methods: Statistical analysis  Page No.4 |
|  | 13e | Describe any methods used to explore possible causes of heterogeneity among study results (e.g. subgroup analysis, meta-regression). | Results  Disease activity based analysis |
|  | 13f | Describe any sensitivity analyses conducted to assess robustness of the synthesized results. | Materials and methods: Sensitivity analysis  Page No. 5 |
| Reporting bias assessment | 14 | Describe any methods used to assess risk of bias due to missing results in a synthesis (arising from reporting biases). | Materials and methods: Assessment of publication bias  Page No.4 |
| Certainty assessment | 15 | Describe any methods used to assess certainty (or confidence) in the body of evidence for an outcome. | Materials and methods: Statistical analysis  Page No.4 |
| **RESULTS** | | |  |
| Study selection | 16a | Describe the results of the search and selection process, from the number of records identified in the search to the number of studies included in the review, ideally using a flow diagram. | Results:  Figure 1 |
|  | 16b | Cite studies that might appear to meet the inclusion criteria, but which were excluded, and explain why they were excluded. | NA |
| Study characteristics | 17 | Cite each included study and present its characteristics. | Results:  Table 1 |
| Risk of bias in studies | 18 | Present assessments of risk of bias for each included study. | Results: Assessment of publication bias  Page No.6 |
| Results of individual studies | 19 | For all outcomes, present, for each study: (a) summary statistics for each group (where appropriate) and (b) an effect estimate and its precision (e.g. confidence/credible interval), ideally using structured tables or plots. | Results:  Table S5 to S12 |
| Results of syntheses | 20a | For each synthesis, briefly summarise the characteristics and risk of bias among contributing studies. | Results: Assessment of publication bias  Page No.6 |
|  | 20b | Present results of all statistical syntheses conducted. If meta-analysis was done, present for each the summary estimate and its precision (e.g. confidence/credible interval) and measures of statistical heterogeneity. If comparing groups, describe the direction of the effect. | Results |
|  | 20c | Present results of all investigations of possible causes of heterogeneity among study results. | Discussion |
|  | 20d | Present results of all sensitivity analyses conducted to assess the robustness of the synthesized results. | Results: Statistical analysis  Page No.10 |
| Reporting biases | 21 | Present assessments of risk of bias due to missing results (arising from reporting biases) for each synthesis assessed. | Results: Assessment of publication bias  Page No.6 |
| Certainty of evidence | 22 | Present assessments of certainty (or confidence) in the body of evidence for each outcome assessed. | Results |
| **DISCUSSION** | | |  |
| Discussion | 23a | Provide a general interpretation of the results in the context of other evidence. | Discussion  Page 11 |
|  | 23b | Discuss any limitations of the evidence included in the review. | Discussion  Page 12 |
|  | 23c | Discuss any limitations of the review processes used. | Discussion  Page 12 |
|  | 23d | Discuss implications of the results for practice, policy, and future research. | Discussion  Page 12 |
| **OTHER INFORMATION** | | |  |
| Registration and protocol | 24a | Provide registration information for the review, including register name and registration number, or state that the review was not registered. | NA |
|  | 24b | Indicate where the review protocol can be accessed, or state that a protocol was not prepared. | Not prepared |
|  | 24c | Describe and explain any amendments to information provided at registration or in the protocol. | NA |
| Support | 25 | Describe sources of financial or non-financial support for the review, and the role of the funders or sponsors in the review. | Funding information |
| Competing interests | 26 | Declare any competing interests of review authors. | Conflict of Interest |
| Availability of data, code and other materials | 27 | Report which of the following are publicly available and where they can be found: template data collection forms; data extracted from included studies; data used for all analyses; analytic code; any other materials used in the review. | NA |

*From:*  Page MJ, McKenzie JE, Bossuyt PM, Boutron I, Hoffmann TC, Mulrow CD, et al. The PRISMA 2020 statement: an updated guideline for reporting systematic reviews. BMJ 2021;372:n71. doi: 10.1136/bmj.n71

For more information, visit: <http://www.prisma-statement.org/>

| **Table S3. Newcastle–ottawa quality assessment scale for selected studies.** | | | | | | | | | |
| --- | --- | --- | --- | --- | --- | --- | --- | --- | --- |
| **Study** | **Selection** | | | | **comparability** | **Exposure** | | | **Total Score** |
| **Abdallah et al.,2009[33]** | * | * | * | * | ** | * | * |  | 8 |
| **Ala et al.,2015 [30]** | * | * | * | * | * | * | * |  | 7 |
| **Bhardwaj et al.,2020[24]** | * | * | * | * | * | * | * |  | 7 |
| **Dwivedi et.al.,2013[8]** | * | * | * | * | ** | * | * |  | 8 |
| **Eby et al.,2015[50]** | * | * | * | * | ** | * | * |  | 8 |
| **Elela et al.,2013[25]** | * | * | * | * | * | * | * |  | 7 |
| **El-Komy et al., 2012[28]** | * | * | * | * | * | * | * |  | 7 |
| **Ghanem et al.,2017[36]** | * | * | * | * | * | * | * |  | 7 |
| **Giri et al.,2020a[5]** | * | * | * | * | ** | * | * |  | 8 |
| **Giri et al.,2020b[22]** | * | * | * | * | ** | * | * |  | 8 |
| **Giri et al.,2021[9]** | * | * | * | * | * | * | * |  | 7 |
| **Hegab et al.,2015[17]** | * | * | * | * | ** | * | * |  | 8 |
| **Hegazy et al.,2014[26]** | * | * | * | * | * | * | * |  | 7 |
| **Huo et al.,2021[19]** | * | * | * | * | ** | * | * |  | 8 |
| **Kalaiselvi et al.,2019[34]** | * | * | * | * | ** | * | * |  | 8 |
| **Kidir et al.,2017[29]** | * | * | * | * | ** | * | * |  | 8 |
| **Klarquist et al.,2010[14]** | * | * | * | * | ** | * | * |  | 8 |
| **Lili et al.,2012[18]** | * | * | * | * | ** | * | * |  | 8 |
| **Lv et al.,2019[49]** | * | * | * | * | ** | * | * |  | 8 |
| **Miao et al.,2018[51]** | * | * | * | * | ** | * | * |  | 8 |
| **Mukhatayev et al.,2020[47]** | * | * | * | * | ** | * | * |  | 8 |
| **Osman et al.,2015[31]** | * | * | * | * | * | * | * |  | 7 |
| **Sohafy et al.,2021[48]** | * | * | * | * | ** | * | * |  | 8 |
| **Taher et al.,2009[52]** | * | * | * | * | ** | * | * |  | 8 |
| **Tembhre et al., 2015[20]** | * | * | * | * | ** | * | * |  | 8 |
| **Tu et al.,2011[32]** | * | * | * | * | ** | * | * |  | 8 |
| **Zhang et al.,2018[21]** | * | * | * | * | ** | * | * |  | 8 |
| **Zhang et al.,2021[53]** | * | * | * | * | ** | * | * |  | 8 |
| **ZHuo et al.,2012[35]** | * | * | * | * | ** | * | * |  | 8 |
| **ZHuo et al.,2015[37]** | * | * | * | * | ** | * | * |  | 8 |
| **Each category can be awarded with one point (^*^).^a^Up to two points can be given to this category (^**^) when additional factors are controlled.** | | | | | | | | | |

| **Table S4. Test for Publication bias.** | | | | |
| --- | --- | --- | --- | --- |
| **Eggers test** | | **Test of Publication Bias** | | |
| **Z value** | **p value** | **chi square** | **df** | **p value** |
| **0.533** | 0.594 | 4.702 | 4 | 0.319 |

| **Table S5. Sensitivity analysis: Standardized mean difference for Treg frequency in vitiligo patients and controls.** | |
| --- | --- |
| **Treg Frequency** | |
| **Abdallah et al., 2009** | 1.02 [0.35, 1.68] |
| **Dwivedi et al., 2013** | -3.69 [-4.26, -3.12] |
| **Hegab et al., 2015** | -1.42 [-1.80, -1.05] |
| **Huo et al., 2021** | -2.86 [-3.92, -1.81] |
| **Kalaiselvi et al., 2019** | 0.11 [-0.20, 0.42] |
| **Klarquist et al., 2010** | -1.67 [-3.08, -0.26] |
| **Lili et al., 2012** | -1.50 [-2.08, -0.93] |
| **Tembhre et al., 2015** | -0.99 [-1.40, -0.57] |
| **Zhang et al., 2018** | -1.80 [-2.26, -1.33] |
| **ZHuo et al., 2012** | -0.08 [-0.50, 0.34] |
| **Total (95% CI)** | **-1.26 [-2.04, -0.48]** |

| **Table S6. Sensitivity analysis: Standardized mean difference for Treg suppressive capacity levels in vitiligo patients and controls.** | |
| --- | --- |
| **Suppression of CD4^+^ T cells** | |
| **Giri et al., 2020b** | -3.72 [-4.38, -3.06] |
| **Klarquist et al., 2010** | 0.52 [-1.16, 2.20] |
| **Tu et al., 2011** | -0.42 [-1.37, 0.53] |
| **Subtotal (95% CI)** | **-1.27 [-4.00, 1.45]** |
| **Suppression of CD8^+^ T cells** | |
| **Giri et al., 2020b** | -4.27 [-4.99, -3.55] |
| **Lili et al., 2012** | -3.34 [-5.61, -1.08] |
| **Zhang et al., 2018** | -3.81 [-4.47, -3.15] |
| **Subtotal (95% CI)** | **-3.99 [-4.47, -3.51]** |
| **Total (95% CI)** | **-2.58 [-3.95, -1.21]** |

| **Table S7. Sensitivity analysis: Standarized mean difference for FOXP3 protein levels in vitiligo patients and controls.** | |
| --- | --- |
| **FOXP3 protein levels in blood** | |
| **Abdallah et al., 2009** | 1.13 [0.45, 1.80] |
| **Dwivedi et al., 2013** | -4.38 [-5.02, -3.74] |
| **Giri et al., 2020a** | -6.25 [-7.26, -5.25] |
| **Giri et al., 2020b** | -33.05 [-37.75, -28.36] |
| **Giri et al., 2021** | -8.21 [-9.10, -7.32] |
| **Hegab et al., 2015** | -0.47 [-0.81, -0.13] |
| **Kalaiselvi et al., 2019** | 0.11 [-0.20, 0.42] |
| **ZHuo et al., 2012** | -0.05 [-0.48, 0.37] |
| **Subtotal (95% CI)** | **-4.94 [-6.96, -2.91]** |
| **FOXP3 protein levels in skin** | |
| **Bhardwaj et al., 2020** | -16.59 [-19.70, -13.48] |
| **Elela et al., 2013** | -10.90 [-12.13, -9.67] |
| **Hegazy et al., 2014** | -5.99 [-7.50, -4.47] |
| **Kidir et al., 2017** | -2.60 [-3.63, -1.56] |
| **Lili et al., 2012** | -0.37 [-2.00, 1.25] |
| **Subtotal (95% CI)** | **-7.16 [-11.73, -2.59]** |
| **FOXP3 transcripts in blood** | |
| **Bhardwaj et al., 2020** | Not estimable |
| **Elela et al., 2013** | -2.19 [-2.58, -1.80] |
| **Giri et al., 2020a** | Not estimable |
| **Huo et al., 2021** | -4.68 [-6.13, -3.22] |
| **Subtotal (95% CI)** | **-3.33 [-5.76, -0.90]** |
| **Total (95% CI)** | **-5.43 [-6.98, -3.88]** |

| **Table S8. Sensitivity analysis: Standardized mean difference for IL-10 protein levels in vitiligo patients and controls.** | |
| --- | --- |
| **IL-10 protein levels in blood** | |
| **Ala et al., 2015** | -0.57 [-0.81, -0.33] |
| **Giri et al., 2020a** | -7.78 [-8.99, -6.57] |
| **Giri et al., 2020b** | -10.01 [-11.48, -8.54] |
| **Zhang et al., 2018** | -3.77 [-4.42, -3.11] |
| **Subtotal (95% CI)** | **-5.48 [-9.20, -1.75]** |
| **IL-10 protein levels in skin** | |
| **Kidir et al., 2017** | -0.57 [-2.27, 1.13] |
| **Taher et al., 2009** | 0.38 [-0.24, 1.01] |
| **Subtotal (95% CI)** | **0.25 [-0.40, 0.90]** |
| **Total (95% CI)** | **-3.68 [-6.10, -1.26]** |

| **Table S9. Sensitivity analysis: Standardized mean difference for TGF-β levels in vitiligo patients and controls.** | |
| --- | --- |
| **TGF-β protein levels blood** | |
| **EL-Komy et al., 2012** | Not estimable |
| **Ghanem et al., 2017** | -0.17 [-0.61, 0.28] |
| **Giri et al., 2020a** | -0.58 [-0.99, -0.16] |
| **Giri et al., 2020b** | -9.74 [-11.17, -8.30] |
| **Osman et al., 2015** | -0.15 [-0.57, 0.28] |
| **Tu et al., 2011** | -1.16 [-1.68, -0.63] |
| **Zhang et al., 2018** | -2.98 [-3.54, -2.41] |
| **ZHuo et al., 2015** | 1.34 [0.88, 1.80] |
| **Subtotal (95% CI)** | **-1.77 [-3.14, -0.40]** |
| **TGF-β protein levels skin** | |
| **EL-Komy et al., 2012** | -0.37 [-1.14, 0.39] |
| **Ghanem et al., 2017** | Not estimable |
| **Kidir et al., 2017** | 0.00 [-0.54, 0.54] |
| **Subtotal (95% CI)** | **-0.12 [-0.57, 0.32]** |
| **Total (95% CI)** | **-1.40 [-2.49, -0.30]** |

| **Table S10. Sensitivity analysis: Standardized mean difference for Treg levels in vitiligo post treatment.** | |
| --- | --- |
| **Treg frequency in vitiligo mice model studies** | |
| **Eby et al., 2015** | 0.96 [-0.48, 2.40] |
| **Miao et al., 2018** | 4.44 [2.00, 6.89] |
| **Mukhatayev et al., 2020** | Not estimable |
| **Zhang et al., 2021** | 1.72 [0.31, 3.13] |
| **Subtotal (95% CI)** | **2.11 [0.43, 3.78]** |
| **Treg frequency in vitiligo Human studies** | |
| **Huo et al., 2021** | 2.12 [1.21, 3.04] |
| **Lv et al., 2019** | Not estimable |
| **Sohafy et al., 2021** | 0.28 [-0.16, 0.72] |
| **Subtotal (95% CI)** | **1.16 [-0.65, 2.96]** |
| **Total (95% CI)** | **1.64 [0.45, 2.83]** |

| **Table S11. Sensitivity analysis: Standardized mean difference foxp3 levels in vitiligo post treatment.** | |
| --- | --- |
| **FOXP3 levels in vitiligo mice model studies** | |
| **Eby et al., 2015** | 2.27 [0.68, 3.85] |
| **Miao et al., 2018** | 4.44 [2.00, 6.89] |
| **Mukhatayev et al., 2020** | Not estimable |
| **Zhang 2021** | 6.58 [0.03, 13.13] |
| **Subtotal (95% CI)** | **3.43 [1.44, 5.43]** |
| **FOXP3 levels in vitiligo human studies** | |
| **Hegazy et al., 2014** | 3.92 [2.82, 5.01] |
| **Huo et al., 2021** | 4.86 [3.36, 6.37] |
| **Lv et al., 2019** | Not estimable |
| **Sohafy et al., 2021** | 1.46 [0.97, 1.96] |
| **Subtotal (95% CI)** | **3.34 [1.13, 5.54]** |
| **Total (95% CI)** | **3.43 [1.90, 4.96]** |

| **Table S12. Sensitivity analysis: Standardized mean difference for IL-10 levels in vitiligo post treatment.** | |
| --- | --- |
| **IL-10 levels in vitiligo mice model studies** | |
| **Mukhatayev et al., 2020** | 2.20 [-0.46, 4.85] |
| **Zhang et al., 2021** | 3.43 [1.41, 5.46] |
| **Subtotal (95% CI)** | **2.98 [1.37, 4.59]** |
| **IL-10 levels in vitiligo human studies** |  |
| **Lv et al., 2019** | Not estimable |
| **Taher et al., 2009** | 1.15 [0.48, 1.82] |
| **Zhang et al., 2018** | 0.65 [0.25, 1.05] |
| **Subtotal (95% CI)** | **0.82 [0.35, 1.29]** |
| **Total (95% CI)** | **1.32 [0.47, 2.17]** |
