# Supplementary material for: Meta-Analysis of Alterations in Regulatory T Cells' Frequency and Suppressive Capacity in Patients with Vitiligo
Source: J Immunol Res. 2022 Sep 16;2022:6952299. doi: 10.1155/2022/6952299 (PMC9508461; doi:10.1155/2022/6952299)
Supplement: Supplementary 2 — Figure S1: the forest plots for FOXP3 expression levels in active vitiligo and stable vitiligo patients and lesional, perilesional, and nonlesional skin of vitiligo patients. (a) FOXP3 protein expression in active vitiligo patients vs. stable vitiligo patients (p = 0.01; SMD: -2.99 [-5.26, -0.71]). (b) FOXP3 levels in lesional skin vs. perilesional skin (p = 0.007, SMD: -9.77 [-16.91, -2.62]). (c) FOXP3 levels in lesional skin vs. nonlesional skin (p = 0.04, SMD: -1.01 [-1.95, -0.06]). Figure S2: the forest plots for IL-10 and TGF-β expression levels in active vitiligo and stable vitiligo patients. (a) IL-10 protein expression in active vitiligo patient's vs. stable vitiligo patients (p = 0.09, SMD: -1.67 [-3.59, 0.25]). (b) TGF-β levels in active vitiligo patient's vs. stable vitiligo patients (p = 0.06; SMD: -3.49 [-7.08, 0.10]). [file 6952299.f2.doc]

**
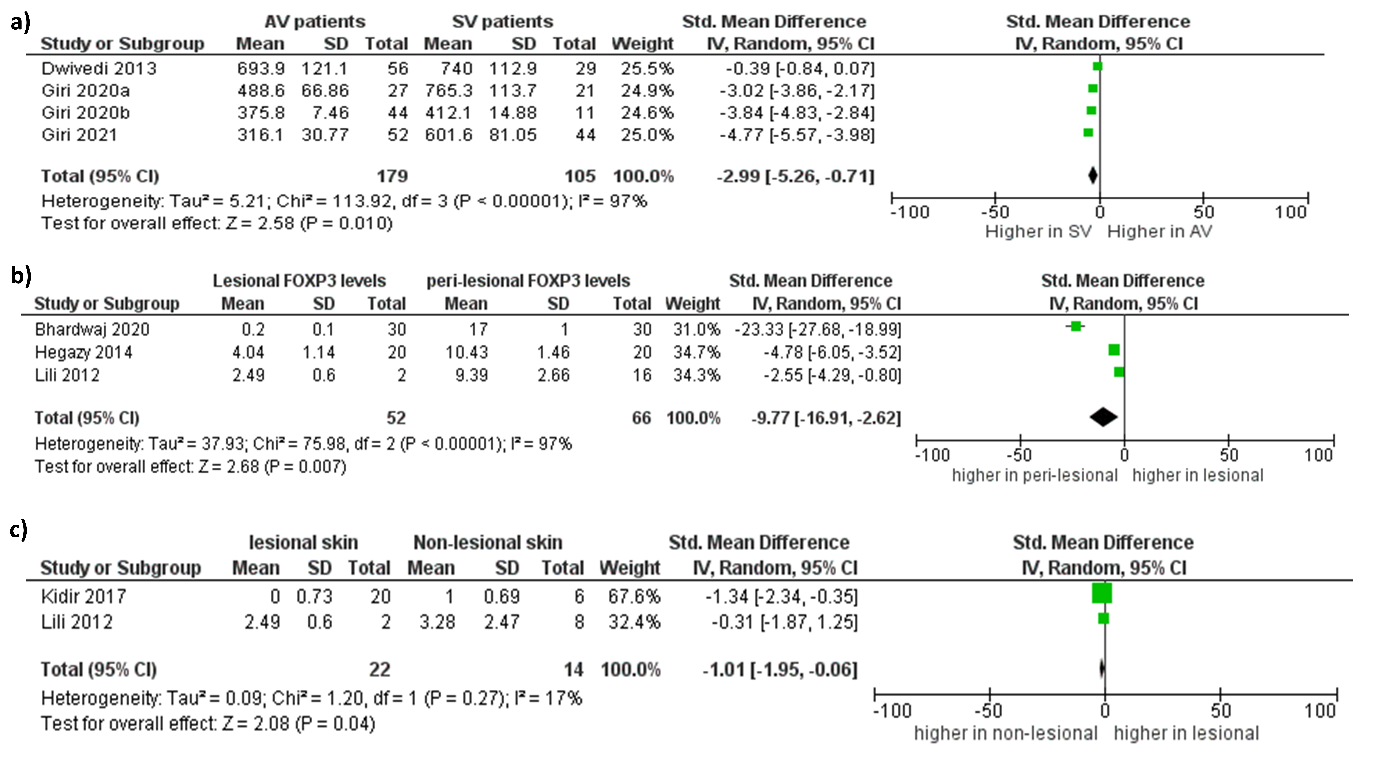
**

**Figure S1. The forest plots for FOXP3 expression levels in active vitiligo & stable vitiligo patients and lesional, peri-lesional & non-lesional skin of vitiligo patients.** (a) FOXP3 protein expression in active vitiligo patients vs stable vitiligo patients (p=0.01; SMD: -2.99 [-5.26, -0.71]). (b) FOXP3 levels in lesional skin vs peri-lesional skin (p=0.007, SMD: -9.77 [-16.91, -2.62]). (c) FOXP3 levels in lesional skin vs non-lesional skin (p=0.04, SMD: -1.01 [-1.95, -0.06]).

**
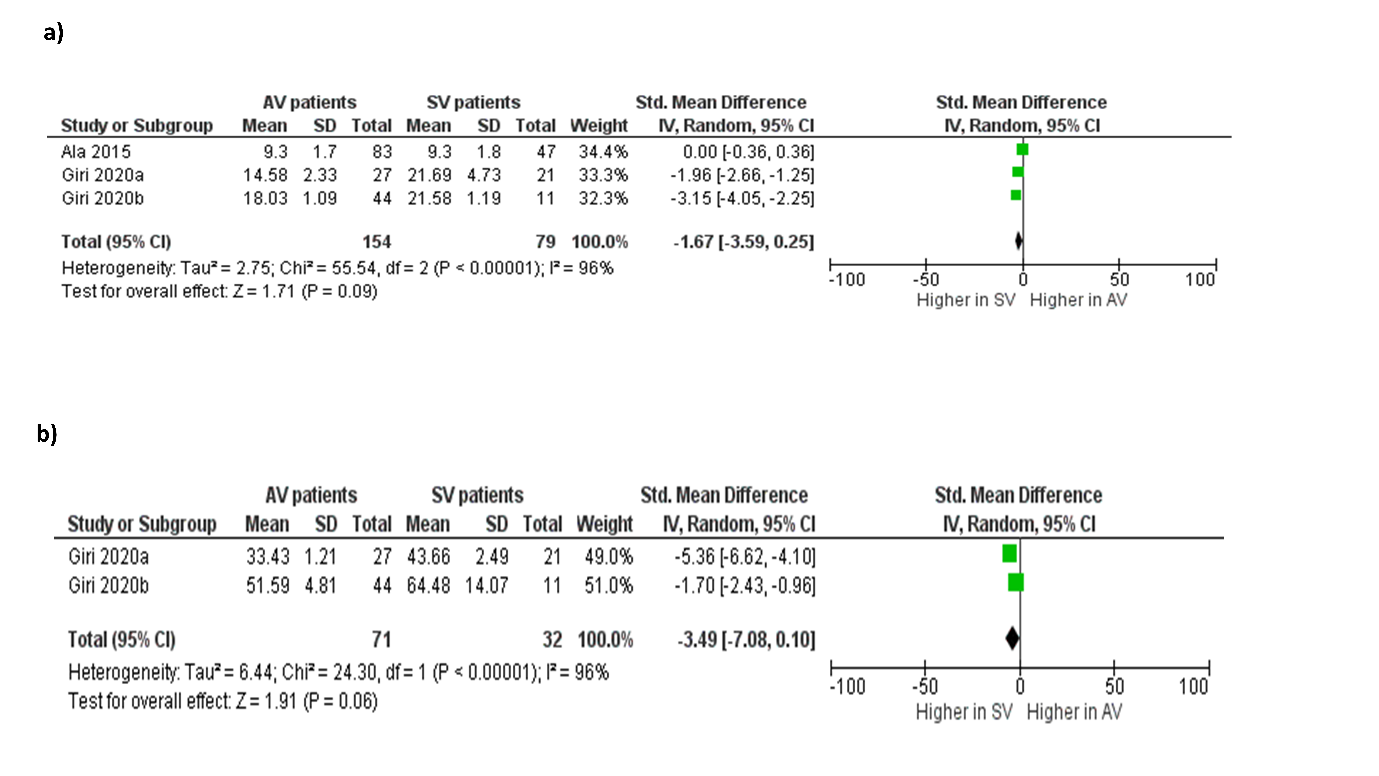
**

**Figure S2.** **The forest plots for IL-10 and TGF-β expression levels in active vitiligo and stable vitiligo patients.** (a) IL-10 protein expression in active vitiligo patient’s vs stable vitiligo patients (p=0.09, SMD: -1.67 [-3.59, 0.25]). (b) TGF-β levels in active vitiligo patient’s vs stable vitiligo patients (p= 0.06; SMD: -3.49 [-7.08, 0.10]).
